# Advances in the Potential Application of 3D Food Printing to Enhance Elderly Nutritional Dietary Intake

**DOI:** 10.3390/foods12091842

**Published:** 2023-04-28

**Authors:** Yisha Xie, Qingqing Liu, Wenwen Zhang, Feng Yang, Kangyu Zhao, Xiuping Dong, Sangeeta Prakash, Yongjun Yuan

**Affiliations:** 1 Chongqing Key Laboratory of Speciality Food Co-Built by Sichuan and Chongqing, School of Food and Bioengineering, Xihua University, Chengdu 610039, China; 2 Academy of Food Interdisciplinary Science, School of Food Science and Technology, Dalian Polytechnic University, Dalian 116034, China; 3 School of Agriculture and Food Sciences, University of Queensland, Brisbane 4072, Australia

**Keywords:** 3D food printing, geriatric food, food appearance, nutrition

## Abstract

The contradiction between the growing demand from consumers for “nutrition & personalized” food and traditional industrialized food production has consistently been a problem in the elderly diet that researchers face and discuss. Three-dimensional (3D) food printing could potentially offer a solution to this problem. This article reviews the recent research on 3D food printing, mainly including the use of different sources of protein to improve the performance of food ink printing, high internal phase emulsion or oleogels as a fat replacement and nutrition delivery system, and functional active ingredients and the nutrition delivery system. In our opinion, 3D food printing is crucial for improving the appetite and dietary intake of the elderly. The critical obstacles of 3D-printed food for the elderly regarding energy supplements, nutrition balance, and even the customization of the recipe in a meal are discussed in this paper. By combining big data and artificial intelligence technology with 3D food printing, comprehensive, personalized, and customized geriatric foods, according to the individual traits of each elderly consumer, will be realized via food raw materials-appearance-processing methods. This article provides a theoretical basis and development direction for future 3D food printing for the elderly.

## 1. Introduction

It is estimated that the global population is aging. Virtually every country in the world has experienced a growth in the number and proportion of older people. According to the data from World Population Prospects: the 2022 Revision, by 2050, one in six people in the world will be over 65 years of age (16%), up from 1 in 11 in 2019 (9%) [1]. China, one of the largest populations in the world, has 264.02 million people over 60 years old and 190.64 million over 65 years old, which is about 13.50% of the overall population [2]. The population aging problem in China will lead to a series of social and economic problems. Ensuring the health, well-being, physical activity, and quality of life of the elderly has become important. Above all, a balanced diet is essential for maintaining a healthy lifestyle [3].

Functional ability deteriorates progressively as people age, and problems with taste, chewing, and swallowing arise. A range of nutrition-impacting symptoms, such as poor appetite, lack of digestion, and inadequate absorption, influence the health of the elderly [4]. Moreover, the major contributing factor to the imbalances in the elderly nutrition is an imbalance in dietary intake, which is the human need, and a poor diet. In view of the poor chewing and swallowing ability of the elderly, the diet texture should be adjusted. Diet texture can be divided into a soft, finely divided, semiliquid, and full-liquid diet. Although liquid diets can be safely swallowed by the elderly, the form of food can be unappetizing. Food design with 3D-printing could resolve this bottleneck.

Three-dimensional printing stands as a novel processing technology in the food industry. The freedom of design allows the user to manufacture novel food products with digitalized intricate shapes, novel textures, and high nutritional value by combining different food ingredients and printing methodologies. Research has revealed that senior consumers, i.e., older consumers, considered the 3D-printed products available from nursing home companies to be appetizing [5]. The 3D-printed products were served to senior consumers either in forms reminiscent of the original ingredients or in exciting shapes, such as flowers [5]. In the study developed by Molitch-Hou [5], the data revealed that elderly consumers were willing to try the new foods, suggesting that research and development of 3D-printed food for the elderly is a major opportunity for the food industry.

In the geriatric food market, the food provided to the elderly is daily and instant food, such as milk powder, oats, and sesame paste. However, the design of these foods is based on the consideration of normal consumers, and they are not suitable for the elderly who really need them. In addition, the “beauty” problem should be considered as well, which reflects the emotional value of the product to consumers. The food provided by 3D printing to elderly has the advantages of soft texture, nutrition, satiety, and visual properties [6], helping the elderly to eat safely and nutritiously while feeling the “joy of eating”. Three-dimensional-printed meals provided to elderly persons with swallowing difficulties has developed very quickly in some countries and regions, indicated that printed food has the capacity to expand and thrive [6]. This paper reviews recent research on 3D food printing, including the use of different sources of protein to improve the food ink printing performance, high internal phase emulsion or oleogels as a fat replacement and nutrition delivery system, functional active ingredients, and the nutrition delivery system. Trends and challenges in expanding the 3D-printed diet range for people with dysphagia and the elderly are also discussed. This reviews provides the theoretical basis and development direction for future 3D food printing for the elderly.

## 2. Particular Texture of 3D-Printing Ink to Eat Safety

Elderly individuals can have oral injury problems such as decreased occlusion force, tooth loss, and reduced salivary secretion, causing discomfort and requiring the intake of texture-modified food [7]. Over the past decades, 3D food-printing technologies have been proposed as a technology-driven solution for providing visually appealing texture-modified foods to those with dysphagia [8]. Extrusion-based 3D printing is the most extensively used since it is successful with a wide variety of food materials [9]. It is essential that the food material or ink used in extrusion-based printing is soft during extrusion and solid once deposited to support the next layer of printing. Food produced with extrusion-based 3D printing may be suitable for the elderly. Research on the provision of 3D printing texture-modified foods for people with dysphagia has focused predominantly on compliance with reducing particle size in solid food or thickening fluid texture to reduce the risk of choking. Physical means, hydrocolloids, or proteins commonly modulate the rheological and sensory properties of texture-modified food. Food particle size could be reduced and modified during the physical process, resulting in proper viscoelasticity. For example, after decreasing the particle size of steamed fish and carrot pulp (CP) using a colloid mill, the fish paste was found suitable to dysphagia patients, and the CP-potato starch–xanthan gum mixture 3D-printing stability improved [10,11].

**Figure 1:**
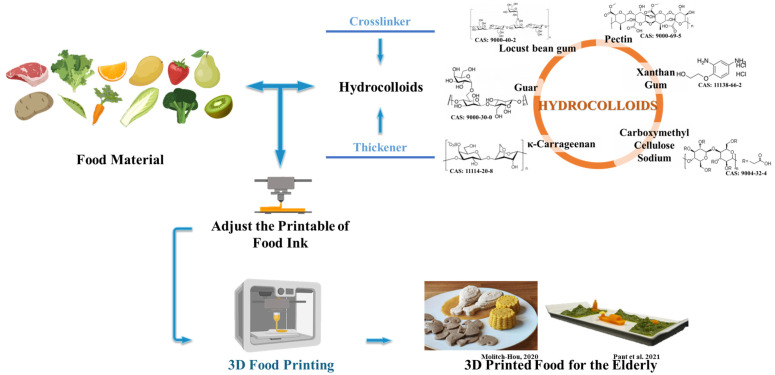
Three-dimensional food printing with hydrocolloids (adapted from Molitch-Hou [5] and Pant et al. [16]) (reproduced or adapted from Molitch-Hou [5], with permission from Molitch-Hou, M. 2020., reproduced or adapted from Pant et al. [6], with permission from Elsevier, 2021).

Hydrocolloids, such as gelatin, pectin, carrageenan, agar, konjac glucomannan (KGM), and alginate, are polymers that can form gels or viscous solutions, which are commonly used in 3D food printing, as shown in Figure 1. The hydrocolloids are commonly used as the modifiers for the 3D printing of food inks, which have been used in various common foods. Hydrocolloids as a thickener has been extensively used in the 3D printing of various fruit juices, such as mango juice concentrate [12], lemon juice [13] orange concentrate [14], and strawberry juice [15]. Fresh and frozen vegetable inks can be prepared by adding hydrocolloids, i.e., xanthan gum (XG), kappa carrageenan (KC), and locust bean gum (LBG) for texture modification, and the printed food has been found suitable for dysphagia patients [16]. Dick and colleagues investigated different hydrocolloids at various ratios to improve the printability of pork and beef meat. The researchers found that XG and guar gum (GG) provided more viscoelasticity and stability to the pork paste for printing, and the printed products were categorized as potential transitional foods within the International Dysphagia Diet Standardisation Initiative (IDDSI) Framework [17]. Yun and collaborators developed a kind of 3D food printing ink containing abalone powder and soybean protein, with gelatin being included to adjust the texture, in order to meet the criteria for senior-friendly foods [18]. Liu and collaborators have realized the printing of shiitake mushrooms with the addition of κ-carrageenan gum (KG), XG, and Arabic gum (AG), which increased the mechanical strength of the self-supporting capacity of inks, and the printed samples were classified as level 5-minced and moist dysphagia diets [19]. Qiu and colleagues found a similar result in the apple and edible rose blends [20]. Mixing XG and basil seed gum at a ratio of 2:1 was feasible for 3D printing and swallowing of the blends, and the 3D-printed products could be divided into a level 5 dysphagia diet. In addition to the above land-based foods, hydrocolloids can also be used in the 3D printing of seafood. The κ-carrageenan improved the gel network of *Hypophthalmichthys molitrix* sea cucumber (HM-CS) surimi samples to be more compact and uniform, resulting in HM-CS surimi suitable for 3D printing [21]. Although we do not suggest here that HM-CS surimi can be used in a dysphagia or elderly diet, results from the literature support the idea that hydrophilic colloids such as κ-carrageenan contribute to the realization of seafood 3D printing. Researchers can choose appropriate types and proportions of hydrocolloids to develop foods for those elderly with swallowing or chewing difficulties.

## 3. Esthetics in 3D-Printed Food for Improving Elderly Appetite

Appetite is regulated by a complex system of interconnected stimuli, such as vision, hearing, touch, and kinesthetics, which the human organism uses to perceive the texture of food during a meal. Increasing the visual appeal of food has been shown to improve a lack of appetite in pilot studies [22,23]. For these reasons, and to create highly acceptable food products that meet nutritional requirements, the mechanisms involved in texture perception have been investigated. Three-dimensional printing technology appears to improve food esthetics by virtue of its ability to produce foods with suitable texture, a greater nutritional profile, and a more pleasing appearance [24]. Dick et al. developed recombinant meat products and showed that these products could be 3D-printed from soft meat paste, lipids, and alternative ingredients to bring them close to the original flavors and nutrients of a beefsteak [25,26]. Additionally, these products complied with the textural attributes of dysphagia. Meanwhile, Kouzani and colleagues (2017) endeavored to find a solution by using pureed tuna, pureed pumpkin, and pureed beetroot, producing visually appetizing foods for dysphagia patients using 3D printing [27].

**Figure 2:**
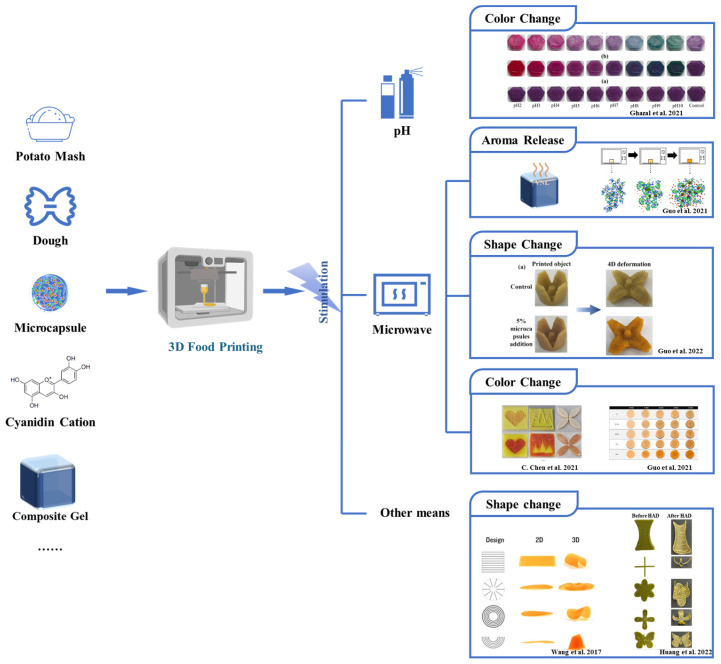
The changes in shape, color, and flavor of printed products triggered by pH, microwaves, and other means to improve appeal and appetite. The image has been adapted from Ghazal et al. [30], Guo et al. [32], Guo et al. [35], Chen et al. [33], Wang et al. [36], and Huang et al. [37] (reproduced or adapted from Ghazal et al. [30], with permission from Elsevier, 2021. reproduced or adapted from Guo et al. [32], with permission from Elsevier, 2021. reproduced or adapted from Guo et al. [35], with permission from Elsevier, 2022. reproduced or adapted from Chen et al. [33], with permission from Elsevier, 2021. reproduced or adapted from Wang et al. [36], with permission from MIT Tangible Media Group, 2017. reproduced or adapted from Huang et al. [37], with permission from Elsevier, 2022).

The application of food processing techniques may trigger 2D-to-3D folding or color change, via processes involving pH, microwaves, hydration-induced wrapping, or temperature-induced self-fragmentation, to present an attractive shape, texture, and aroma (as shown in Figure 2). Research has shown that color, as an important sensory parameter of food, can promote people’s appetite, which is conducive to digestion and absorption, directly affecting consumers’ acceptance and food selection [28,29].The 3D-printed product, made from a mixture of red cabbage juice (rich in anthocyanidin), vanillin, potato starch. and various fruit juices, was found to change its color and flavor in response to external and internal pH stimuli, which improved food products with desired sensory characteristics [30]. There are natural anthocyanins in red cabbage and purple sweet potato. Lin and collaborators found a similar result in 3D-printed peanut protein-polysaccharide hydrogels, which changed color in response to pH changes via addition of a natural pigment [31]. Microencapsulation has been widely used to enhance the flavor or other sensory characteristics of food. A study reported the behavior of color and aroma of 3D-printed buckwheat dough [32]. As microwave treatment is better for the shape stability of 3D-printed products compared to traditional cooking methods, ultrasound was used as an external stimulus after the 3D-printed product was made. During the microwave heating of 3D-printed products, the structure of the microcapsules was gradually destroyed, releasing the pigment and oil, and the concentration of cinnamaldehyde increased. Notably, the addition of microcapsules exerted no significant effect on the printing performance of the buckwheat dough. Chen and collaborators found that the color change of curcumin lotus root gel printed by 3D-printing could also be realized via microwaves l [33]. Similarly, soybean protein isolate (SPI) gel with added carrageenan and vanilla flavor (VNL) can be used for 3D printing, the flavor of the printed product automatically being changed when triggered by microwaves [34]. In the future, users can customize food shape transformations through a predefined simulation platform and produce these designed patterns using 3D food printing. Exploring new stimulation means, such ultrasound or infrared, to induce color and aroma changes of 3D-printed food material is the development direction for making the elderly diet more appetizing.

## 4. Nutritional Composition for a Balanced Diet for the Elderly

For the entire creation process of customized foods, it is necessary to use materials that are large enough to satisfy all consumer requests or small enough to allow for combination in varying proportions, permitting food distribution in a personalized manner to satisfy nutritional needs [38,39]. For basic 3D-printed food, hydrocolloids improve the printing performance of the material but limit the nutritional value of the food to a certain extent. For the elderly, who need more nutrients for their nourishment, the types and contents of nutrients in 3D-printed food need to be further improved. To meet the requirements of protein, lipids, and carbohydrates for the elderly, many researchers have added protein from various sources, emulsion, or dietary fiber to alter the food microstructure and composition for the production of the elderly diet.

### 4.1. Protein Supplementation from Various Sources

Protein can provide energy to patients, especially those elderly who are malnourished, to maintain body weight and muscle mass [9], which can be compatible with 3D food printing (as shown in Table 1). In one study, various prototypes of meat substitute were prepared using protein powder-based materials with different processing parameters on the particle surface, and the texture was evaluated by comparing and analyzing the physical properties [40].

As a whole food, eggs contain egg yolk (EY) and egg white (EW). The properties of the material supply for printing EW and EY are different due to differences in lipids, proteins, and water content. It was found that the lipid fractions of EY act as a plasticizing agent, greatly enhancing the fluidity of rice flour by providing flowability and improving the printing behavior [48]. Nutritionally and functionally, egg white protein (EWP) is a valuable ingredient. These properties of EWP make it a promising material for developing 3D-printed foods, and researchers have achieved complex 3D-printed objects containing egg white protein (EWP), gelatin, cornstarch, and sucrose [39]. The researchers claim that 3D printing based on the EWP mixture system is a promising method for producing complex-shaped food objects due to the good correlation between viscosity and sensory evaluation score. Other researchers have confirmed that EWP has the ability to maintain complex 3D geometries, thus improving the quality and efficiency of 3D printing [49,50]. EWP can potentially be used in 3D food printing for the elderly and may also improve protein intake. 

Whey protein (WP) is derived from milk. In addition to several essential amino acids, WP also contains various essential trace elements, such as calcium, potassium, magnesium, and iron [51]. The mixture of whey protein isolate and milk protein concentrate results in protein paste that has the suitable viscosity and mechanical strength for deposition and adhesion to be applied in extrusion-based 3D-printing [44]. The addition of whey protein could also change the structure of the konjac gel, affecting the performance of the printed products and the fluidity of the extrusion process [51]. Furthermore, as the inner filler, whey protein isolate has the effect of softening, improving smoothness, and reducing the lumpy fracture texture perception, which can be served as an appetizing and protein-rich snack for specific consumer groups with varying dietary requirements [45]. Similarly, the combination of WPI with gelatin was shown to improve the physical properties and printability of yoghurt, with WPI primarily influencing the taste and flavor of the protein-rich yoghurt gels [46].

The proteins in plants, such as oats and peanuts, are different from animal protein or egg proteins in amino acid composition and molecular structure [52]. The nutritional value of the original product can be improved by adding plant proteins. Additionally, plant protein-based materials possess attractive thermomechanical properties, which make them prospective sources for use in 3D printing [53]. Proteins derived from peas have low allergenicity and are rich in essential amino acids (tryptophan and lysine) [41]. In one study, the printability of the banana matrix was improved by increasing the entanglement between the banana matrix and the pea protein [41]. The addition of protein was beneficial in increasing the modulus and adhesion force. On the contrary, the recovery and elongation at the break of the banana-based printing ink decreased. Based on the soft texture of bananas, the 3D-printed products made by banana matrix and pea protein have the potential value of 3D printing ink for the elderly diet. Feng and collaborators found a similar result in that the addition of pea protein improved the cross-linking degree of the granularity, the cohesiveness, adhesiveness, and thermal properties of the potato starch-based 3D-printied materials [54]. The addition of pea protein isolate was also shown to be beneficial for improving the percentage of composite flour to 21.04% [55]. Soy protein isolate (SPI), as one of the most common plant proteins, is already widely used in 3D food printing, with good physicochemical and functional properties [56,57]. Recently, SPI and oats have been formulated as printing materials, realizing the shape change of a 3D printed-model under microwave heating [58]. In similar fashion to SPI, the addition of rice protein was found to reduce the viscosity and soften the edible inks (soy protein- and gluten-based pastes), forming meat analogs under an air-heating extrusion-based 3D printer [59]. In summary, these plant-based inks prepared by mixed protein are useful for 3D food printing.

As a new protein source, insect protein, possessing essential micronutrient and functional biological properties, has been used for food additives and meat substitution agents [47]. As early as 2018, Severini and collaborators studied modifying the printability of wheat flour dough by substituting it with different amounts of yellow mealworm powder [60]. After adding 20 g/100 g (d.b.) of insect, the soft-texture dough was obtained, and the protein digestibility-corrected amino acid score of the dough increased by 23.6 compared with the noninsect-enriched sample. Chao and collaborators used mealworm protein to decrease the viscoelastic properties of chicken surimi ink to improve printing shape retention [47]. The hardness of the printed mealworm protein isolate content-incorporated chicken surimi sample was shown to have a lower hardness value, which is considered suitable for the soft solid diet of elderly individuals. Scheele and collaborators provided the highest fidelity prints of mashed potatoes by adding protein-rich cricket and pea protein powders [61]. A 3D-printed food based on wheat flour with insect (*Acheta domesticus*) powder was shown to improve the aesthetic quality and reduce consumer apathy [62]. These studies demonstrate that the addition of protein increases the shape fidelity, printability, and nutritional profile of 3D-printed food, providing rich strategies for meeting protein needs and health of the elderly.

### 4.2. The Healthy Low-Fat, Low-GI Diet

With an improvement in living standards, an increasing number of elderly persons are becoming concerned about their health. Hyperglycemia, hyperlipidemia, and hypertension are the major chronic diseases in middle-aged and older adults. According to the recommendations of the Chinese Dietary Guidelines [63], the reduction of animal fat intake should be accompanied by increased protein intake. Health-conscious consumers are increasingly demanding low-fat and cholesterol-reduced products. In meat and fish products, one promising approach to reducing fat content involves the addition of emulsion or inulin as a fat replacer, which can load active ingredients that may provide a further health benefit. Shahbazi and collaborators developed a reduced-fat emulsion gel based on soy protein with different biosurfactants (acetylated starch, octenyl succinic anhydride starch) for application in the 3D-printing process [64]. The inulin gels are potential materials for fat analogs because of their fat-like structures [65], which are 3D printable as the emulsion-filled gel of potato starch and inulin. The fat analogs made by coconut oil and soybean oil have the properties of the printability and meltability similar to those of commercial beef and pork fats, which can be utilized in forming marbling patterns in meat analogs [65]. In another study, the 3D-printed acetylated microcrystalline cellulose was examined as a fat analogue in developing low-fat cheese [64]. It exhibited the temporal perception of creaminess, mouth-coating, and fattiness aftertaste, as well as other excellent sensory sensations, showing the potential for the reduced-fat 3D-printed cheese conceived as cheese analogue product. In summary, it is necessary to produce reduced-fat or fat-analogue 3D-printed products with desired functional features and sensory properties that meet the specific requirements of consumers, as in the case of personalized nutrition or for advanced texture perception. This can provide the technology foundation toward tailor-made personalized nutrition according to the specific health condition of the elderly individual.

In the elderly, the blood glucose level is a vital health metric. The glycemic index (GI) is used to classify carbohydrate-based foods according to their glucose response 2 h after consumption and is expressed as a value relative to that of white bread or glucose [66]. Rice, rich in easily digestible carbohydrates, is a type of high-GI food. The structural modification of starch with physicochemical technology, the combination of rice with some of the foods that have a lower GI and higher resistant starch content have been reported to be the main leading approaches for reducing the glycemic index of food. Liu and collaborators reduced starch digestibility by controlling the interaction between starch and glycerol monostearate/stearic acid, enhancing enzymatic resistance within the hot-extrusion 3D-printing environment and maintaining blood glucose concentration at a constant basal level [67]. The health benefits of the functional ingredients of guava, green tea, and barley sprouts include their antioxidant and anticancer effects, which can also help manage chronic hyperglycemia. One study added these functional ingredients to a 3D-printed cheesecake, and the GI index of the functional cheesecake was 35.23–56.49%. indicating that they have a high potential for use as dessert ingredients in low-GI 3D-printed food. The highest total polyphenol content indicated that the green tea cheesecake had excellent antioxidant properties, which was approximately 5–7 times higher than that of other cheesecakes. Based on the fact that all functional powders may improve the printing performance for cheesecake, a 3D-printed functional cheesecake could be an alternative to the production of traditional foods [68].

### 4.3. Nutraceutical and Functional Supplements

An increasing number of researchers are designing 3D-printed food by adding functional and nutraceutical ingredients, focusing on potential foods for health improvement [38]. Products such as the delivery system of functional, active ingredients may be used to promote the health of the elderly.

#### 4.3.1. The Oil-in-Water Emulsion Gel and Oleogels

**Figure 3:**
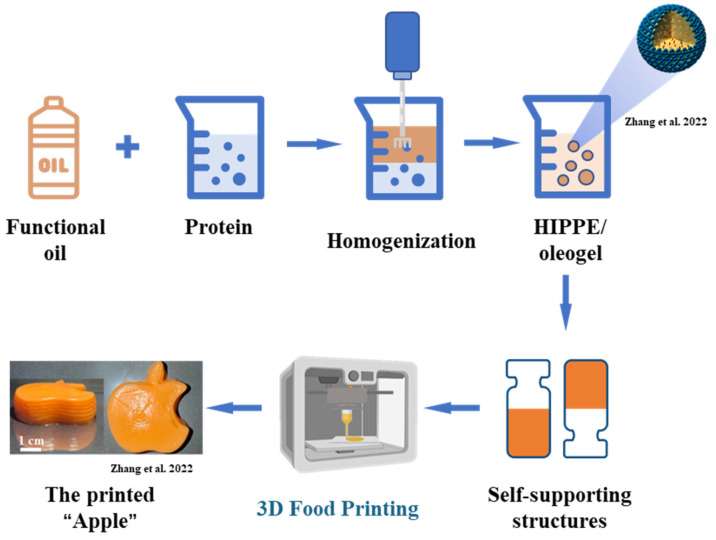
Schematic illustration of the HIPPE or oleogel fabricated by protein or cellulose application in 3D printing. This image has been adapted from Zhang et al. [70] (reproduced or adapted from Zhang et al. [70], with permission from Elsevier, 2022).

Emulsion gels or oleogels may contain and protect naturally occurring bioactive substances, and, as they have the property of viscoelasticity, may have application prospects in 3D-printed food for the elderly. Kavimughil and collaborators investigated the 3D printing of emulsion-templated oleogel developed from gelatin and gellan gum, carrying the lipophilic curcumin and resveratrol as personalized nutraceutical carriers [69]. The oleogel was obtained with 3D printability and stability due to the tight packing of the gellan gum in the lipid phase. In one study, the oil-in-water high internal phase Pickering emulsion (HIPPE) with high oil loading was prepared using protein microgel particles or cellulose as a stabilizer and showed good potential in 3D printing (as shown in Figure 3). The encapsulation of HIPPEs has the effect of maintaining the stability of astaxanthin in an extreme chemical environment as a nutrient delivery system to improve the functionality of the elderly diet [70]. The work of Qiu and collaborators described a similar result: the Pickering emulsion gel prepared using glycyrrhizic acid–zein composite nanoparticles could be beneficial in producing innovative and healthy 3D-printed products containing bioactive ingredients [71].

#### 4.3.2. Functionally Active Ingredients and Nutrition Delivery

**Figure 4:**
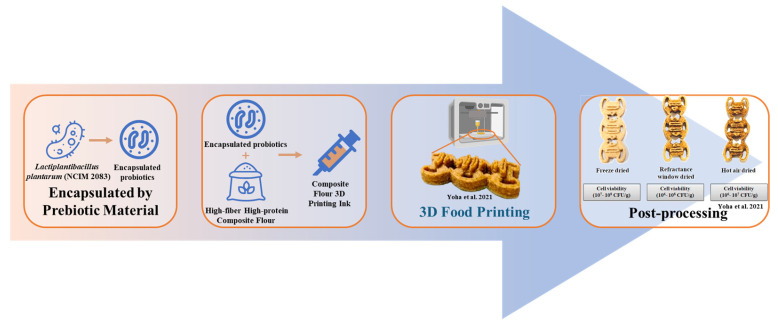
Synergistic encapsulation and 3D printing for improved probiotic stability. This image was adapted from Yoha et al. [77] (reproduced or adapted from Yoha et al. [77], with permission from Elsevier, 2021).

With the continuous development of technology, the concept of the convergence of multiple disciplines and technologies from different industries, such as medicine and biology, has been applied in food science. By combining molecular gastronomy and 3D food printing, functional nourishment will be added into normal food, enhancing the inner biological activities, nutritional profile, and the appearance of the food [24]. Vitamin D insufficiency can lead to a weakened bone matrix and inferior collagen synthesis, resulting in osteoporosis in the elderly. In one study, ergosterol was incorporated into purple sweet potato paste and converted into vitamin D_2_ via UV-C irradiation of 3D-printed potato paste product [72]. Moreover, our group has enhanced the nutrition of a 3D-printed cod protein composite gel by adding flaxseed oil, inulin, and soybean dietary fiber [73]. Based on the previous stage of 3D printing research, 3D-printed cookies have been fortified with the microalga *Arthrospira platensis* or polyphenols extracted from the grape skin, which have been shown to enhance the antioxidant activity of cookies [74,75]. It was found that there was synergism in the antioxidant activity and printing ability of cookies among the phenolic content, printing layer, and infill ratio of 3D models. In addition, some attempts have been made to create 3D-printed food that contains probiotics to alleviate digestive problems associated with gastrointestinal disorders [38]. The feasibility of incorporating *Bifidobacterium animalis* subsp. Lactis BB-12 into 3D-printed smashed potatoes was investigated by Liu and collaborators [76]. The probiotic strain is essential to gastrointestinal health and immune function. The results showed that it was feasible to enrich foods with beneficial micro-organisms, and the bacterial viability in the best treatment was higher than the recommended dose in probiotic foods. Another work reported that a 98–99% survival rate was attained for all the probiotics encapsulated by prebiotic material after the 3D-printing process, and it was evident that the 3D-printing process of high-fiber high-protein composite flour had no negative impact on the viability of encapsulated probiotics (as shown in Figure 4) [77]. Xu and collaborators, aided by the printable property of Pickering emulsion (PE) gel and the potential of protein-stabilized PE being effective in protecting probiotics, were able to stabilize *Bifidobacterium lactis* by incorporating tea protein/xanthan gum [78]. Furthermore, it is promising to produce functional foods using the combination of 3D printing with the encapsulation of probiotic bacteria using protectants or new thermal processing technology such as microwaves. Zhang and collaborators produced baked food with a 10^6^ CFU/g of probiotics viability displaying a health-accelerating effect [79]. Based on our and other authors’ findings, food produced using 3D printing should be nutritionally optimized to meet the special dietary needs of consumers in the future.

## 5. Trends in the Development of 3D Food Printing for the Elderly

A personalized appearance customization feature is an advantage of 3D printing, but printed food has some limitations in personalized nutrition customization. Therefore, the development of 3D food printing should continue to enhance the nutritional aspects of printed food. With the help of the attractiveness of food appearance, the food intake of the elderly can be improved to avoid the occurrence of malnutrition and other related diseases.

### 5.1. Energy Supplementation

Malnutrition is a significant health concern affecting about 10–50% or more older adults [80]. The incidence of malnutrition-related diseases can be reduced by designing meal modules tailored to elderly demands [81]. The nutritional customization of the elderly diet through 3D food printing should be based on protein enrichment, fat replacement, and other nutrition.

#### 5.1.1. Protein Enrichment

Malnutrition is more prevalent among older adults, but this can be reduced by providing a positive energy balance for both total calories and protein [82]. A target of 25–30 g of protein per meal should be achieved at multiple feeding occasions to promote muscle protein synthesis in the elderly, and it cannot be ignored that protein supplementation affects the appetite and energy intake of middle-older aged adults [83]. The use of protein-enriched supplements, such as high-energy drinks packed with carbohydrates and whey protein (a major protein source in dairy), is a common strategy to increase energy intake and body weight in undernourished older people [84]. However, a few researchers focusing on 3D-printed foods have stated that the protein content of their products meets the requirements of the elderly. As a supplement to food, whey protein improves muscle protein synthesis in older individuals [85]. More researchers have used whey protein to improve the viscoelasticity and printability of materials [46,51]. The 3D-printed diets for the elderly are expected to move toward a greater demand for high-quality protein foods, supplied by eggs, milk, fish, and even insects in the future.

#### 5.1.2. Fat Reduction

Malnutrition may coexist with other conditions such as being overweight, and a higher percentage of fat in the diet (energy percentage) is associated with a higher energy intake, leading to higher body weight and representing a double burden among older adults [86,87]. The findings of Wilson and collaborators indicated that high-fat nutritional supplements could suppress the elderly individuals’ appetite [88]. An investigation of 725 Swedish men and women aged 53–80 years by Soderstrom and collaborators suggested that preventive actions to counteract malnutrition in older adults should focus on controlling the intake of total fat in the diet by increasing the consumption of foods with a high content of saturated and monounsaturated fat [86]. Using the emulsion gel, oleogel, which is made from plant oil rich in unsaturated fatty acids, to replace saturated fatty acids in food can benefit consumer health, particularly the elderly. It has been confirmed that gel-like emulsion could be fabricated into delicate shapes using extrusion-based 3D food printing and used in various applications such as meat analogues, cake decoration. or customized functional foods. As the common ingredient in oil-in-water emulsion or oleogel, protein plays a role in maintaining the stability of the interface. Protein is susceptible to heat denaturation, decreasing the ability to maintain the stability of the interface. The heat treatment commonly used in food processing is beneficial to ensuring food safety, but it also increases the incidence of demulsification. Research found the storage modulus (G′) and loss modulus (G″)–temperature curves of the Pickering emulsion gels using complexes of fiber polysaccharide-protein started to decrease at 25 °C and reached a minimum value at around 42 °C, indicating that the emulsion structure of the samples was damaged by temperature [89]. Moreover, the secretion rate of bile salts slows down in the elderly, which affects lipid digestion and absorption. Whether emulsion gel or oleogel with printing properties can satisfy the digestive system of the elderly is a question that deserves further study. Based on this, the positive development of high internal phase emulsion gel and oleogel with digestive stability and heat stability should be pursue in 3D food printing for the elderly.

#### 5.1.3. Nutrition Balance

The design of food structures can be tailored to meet the needs of the elderly depending on the intended use; these approaches are shown in Table 2 [90]. Those elderly individuals with poor glucoregulatory control should reduce the proportion of their daily energy intake provided by carbohydrates. Lower-glycemic-index (GI) foods, such as lotus root, glutinous rice, and buckwheat, cause lower peaks and fewer fluctuations in postprandial blood glucose levels, which are more suitable for the elderly. Modulating starch digestibility could reduce the negative effects of an excessive glucose load while satisfying the energy demand in the elderly. In addition, indigestible carbohydrates, including inulin, oligosaccharides, β-glucans, are considered dietary fibers, and are derived from flours of different cereals and pulses, konjac glucomannan. and other polysaccharides and hydrophilic colloids, which could be considered in the design of 3D-printed food for the elderly. Reports have confirmed that dietary fiber can realize the 3D printing of food [43,73]. The research efforts on 3D food printing for the elderly should be continually strengthened in this direction.

Compared to macronutrient deficiencies, micronutrient deficiencies and low intake of bioactive compounds may be more of a health risk to the elderly than they realize. Designing 3D-printed foods enriched with vitamins, minerals, and bioactive components could be a method to combat micronutrient deficiencies in the elderly. Further, 3D-printing technologies have been used to prepare drug delivery devices. Triastek, Inc., a Chinese company operating in 3D-printing technology, has pioneered melt extrusion deposition (MED) technology. The MED 3D-printing platform allows for the customized design of tablets with different shapes and geometries, controlling drug onset time, duration, and drug interactions with the body. Two medicines have been produced by Triastek, which have received investigational new drug (IND) clearance from the U.S. Food and Drug Administration (FDA) [94]. In the in-depth development and exploration of personalized, customized food for the elderly, we can focus on the nutritional needs of the elderly and discuss the nutritional fortification and delivery according to the drug delivery devices. The future food will ensure that the elderly receive more balanced nutritional elements, promoting human immune function and resistance.

### 5.2. Food Flavor Regulation

An individual’s health goes beyond the absence of disease. The healthy body also includes quality of life (QOL), defined by the World Health Organization’s constitution as aspects of social well-being and maintenance of complete physical and mental modalities [95]. Low appetite could lead to elderly malnutrition, even deriving secondary diseases, which further diminishes their QOL [96]. The taste, smell, and chemesthetic sensation constituting food flavor may be crucial influence in the amount of food intake and the degree of satiety [96]. It has been reported that the patients living in hospitals or nursing homes wanted to eat more food once the flavor of the food improved leading to increased nutrient intake and body weight increase, as well as the health of the elderly individuals [97]. In the future, the aroma and taste of 3D-printed diets for the elderly need to be improved. At the same time, avoiding the addition of ingredients or producing foods with pungent flavor is necessary, which will indirectly improve consumers’ appetites. Presently, 3D-printed food with a soft texture meets the needs of the elderly, whose oral processing and digestion abilities are not as good as those of healthy adults. Based on the oral processing and digestion simulation system of the elderly, researchers can investigate the chewing and digestion characteristics of 3D-printed food using the system and optimize the processing parameters to meet the oral digestion needs of the elderly. This is one of the development trends of 3D-printed food for the elderly.

### 5.3. Nutritional Balance and Control

**Figure 5:**
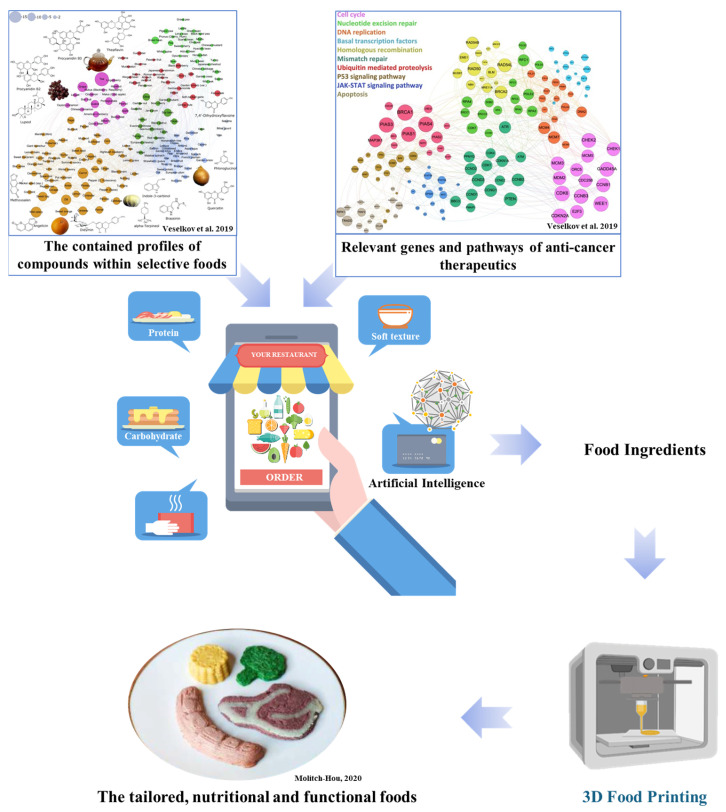
Schematic diagram of food ingredient design and 3D food printing. Adapted from Veselkov et al. [98] and Molitch-Hou [5]. (reproduced or adapted from Veselkov et al. [98], with permission from Springer Nature, 2019. reproduced or adapted from Molotch- Hou [5], with permission from Molitch-Hou, M. 2020).

Based on the above, regarding the addition of protein from various sources, fat substitutes are beneficial to elderly health. The human diet has thousands of bioactive molecules that modulate metabolic and signaling processes, drug actions, and gut microbiota interactions. However, there is a traditional idea in the East, the phenomenon of mutual generation and mutual restriction among the food components, with a synergetic or opposing effect with each other. Researchers have focused on exploring the processing technology of 3D-printed diets for the elderly population’s health, based on the collected big data. The big data include the functions of foods, the background data of the elderly, primary diseases, living habits, the diversity of the intestinal flora, and other information. According to the health database or the wishes of the elderly, accurately investigating the preferences of the elderly consumer groups for different types of food aids in developing the food tastes favored by the elderly. Based on this, by exploiting artificial intelligence (AI) technology, with the multiplying effect of dietary nutrients, calculating the optimal ratio of ingredients, producing special diets with apposite cooking parameters, leveraging the effects of ingredient combinations, high-quality and meticulous care for the elderly will be realized. Veselkov and collaborators have shown that plant-based foods such as tea, carrot, celery, orange, grape, coriander, cabbage, and dill contain the largest number of molecules with high anticancer properties similar to existing therapeutics (as shown in Figure 5) [98]. The machine learning strategy described by the authors was a first step toward realizing the potential role of a “smart” nutritional program in preventing and treating cancer. This methodology is not restricted to cancer and can be applied to other health conditions in the elderly. Yoo and Park have used AI machine learning to obtain adequate print quality in 3D food printing. Through AI machine learning reinforcement learning, print aptitude can be evaluated through rheological analysis, and big data values of various food groups applied with standard composite materials can be secured [99]. In summary, through smart 3D food design for daily life based on big data in health and AI, producing tailored and therapeutically functional foods, personalized customization of meals, and adjustment of nutritional functions can be realized and serve the important needs of elderly health.

## 6. Challenges in 3D Food Printing for the Elderly

As described above, 3D-printed food is beneficial to the elderly in many ways. Most printed food is ready-to-eat food, which is in contrast to the particular consumption habits of the Asian population, especially in China, where people prefer to eat warm food. This habit has driven the interest in printed food that can be eaten between 5 and 60 °C [100], the optimal intake temperature for food and people’s oral cavity, and in maintaining the appearance and texture changes in food for the elderly within an acceptable range. This poses new challenges for printing inks and printing technology. The printing ink should have fluidity and thixotropic properties at the printing temperature to be extrudable. Self-support is also essential for the food ink in achieving layer-upon-layer accumulation after extrusion. In the process of food heat treatment, protein denaturation is accompanied by water transfer and loss, thermally reversible gel melting, and other phenomena, and, with the self-support performance of ink diminishing, this can result in the collapse of the printed product. Therefore, the ingredients of 3D-printed food ink for the elderly, such as hydrocolloids and protein, need to be highly stable during the heat treatment process to produce stable 3D-printed food products in the future.

Thus, there are challenges for researchers focused on food processing and mechanical technology to modify the printing machines or heat treatment means. Some advances in this direction are underway. Researchers have combined microwave equipment and a 3D printer to realize the “print-forming self-gelation” process, which improves the molding quality by enhancing the solid-like behavior of fluid surimi with the synergistic effect of transglutaminase [101]. The highest temperature achieved during extrusion was reported to be about 60 °C, which is in the range of consumer acceptance. In addition, other curing methods, such as selective laser sintering, can be used in 3D food printing.

## 7. Conclusions

Three-dimensional food printing has attracted considerable attention for producing customized food. There are almost limitless possibilities for the 3D printing of food when it comes to soft food for the elderly. Protein, one of the main ingredients in 3D food printing, could improve the protein content of printed products. The viscoelasticity of oil in water emulsion gel or oleogel can be used not only as the fat substitute but also as the delivery system to improve the content of fat-soluble nutrients in printed food. The most promising feature of 3D food printing is the visual appeal of personalized customization. Visual and gustatory appeal can be improved under the stimulation of certain external conditions, raising appetite of older adults. However, some more critical obstacles of 3D-printed food for the elderly should be solved regarding energy supplements, nutritional balance, and recipe customization in a meal, meeting the digestive and absorptive capacity for the elderly and facilitating the absorption of nutrients in the small intestine. In addition, for Asian populations who prefer to eat warm food, can the soft food produced by 3D printing withstand heat treatment and maintain stable quality? In the future of food research and development, we can attempt to integrate multiple disciplines, such as big data in health and AI technology, to help answer the pressing questions. According to the individual elements of each elderly consumer, such as physical condition, dietary preferences, and oral processing ability, the comprehensive, personalized customization of 3D-printed foods can be realized in terms of food raw materials-appearance-processing methods. The nutritional supplement effect of food can be enhanced, and the health level of the elderly can thus be improved.

## Figures and Tables

**Table 1:** Protein supplement of 3D food printing from various sources.

Source	Food Base	Physical Properties	3D Printable	Reference
Egg white protein	A complex system containing egg white protein, gelatin, cornstarch, and sucrose	The heat-induced egg white gel, gelatin, and gelatinized starch cross-linked complex gel network had excellent shape-retention properties.	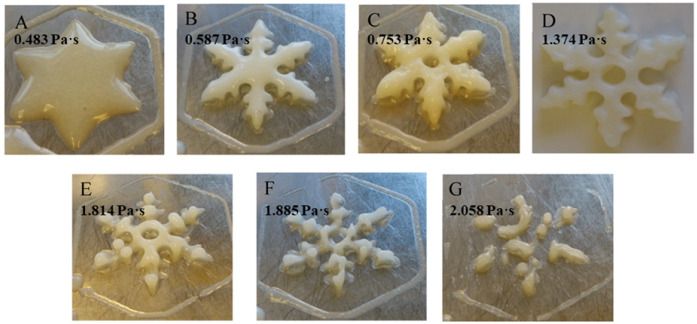	[39]
Plant-based protein: pea protein	Fruit–banana matrix	The incorporation of pea protein isolate increased the entanglement between the banana matrix and the protein, increased the storage modulus and adhesion force, and improved the tailing effect of ink.	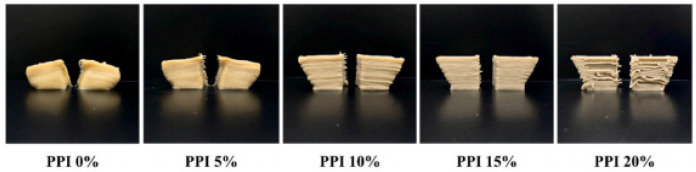	[41]
Plant-based protein: peanut protein	Fruit and vegetable powder	The rheological properties and taste of the peanut protein and fruit/vegetable 3D-printing ink and product were better than those of other normal proteins, such as whey protein isolate, pea protein, casein, and wheat hydrolyzed protein.	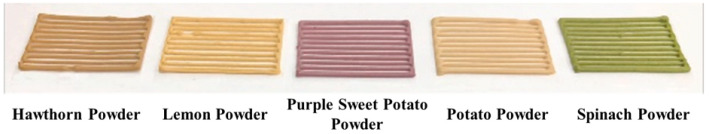	[42]
Plant-based protein: faba bean protein	-	The protein, starch, and fiber mixture of faba bean was a nutritious 3D-printing ink. The protein-rich printed products became clayey after chewing by the tasters.	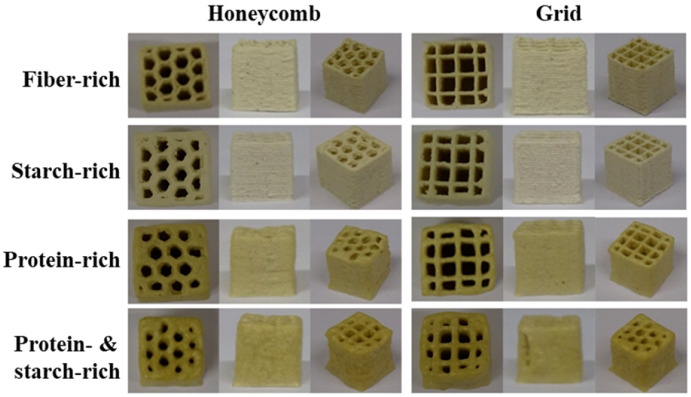	[43]
Whey protein isolate	Milk protein concentrate	The addition of WPI altered the interaction molecules between water and protein, increasing free water and partially immobilized fractions, and reducing ink viscosity. This would benefit the mixture of protein pastes in extrusion-based 3D printing.	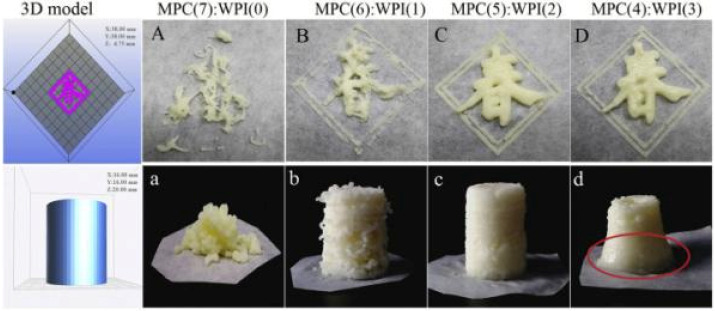	[44]
Whey protein	Konjac gel	The addition of whey protein enhanced the G′, G″, and viscosity of the konjac gel, increasing the density of gel, which improved the supporting performance of the printed products and the fluency of the extrusion process.	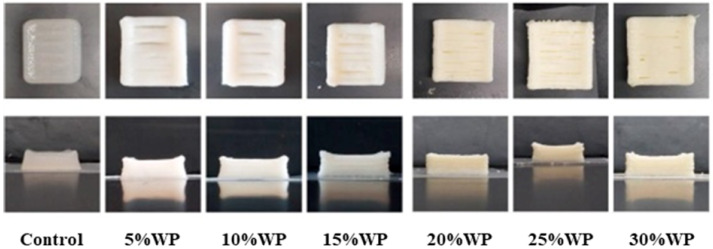	[24]
Whey protein isolate	Lemon mousse	As the inner filler, the whey protein isolate had the effect of softening, improving the adhesiveness of the lemon mousses. The printed mousses added by WPI exhibited well-defined layers and a glossy surface.	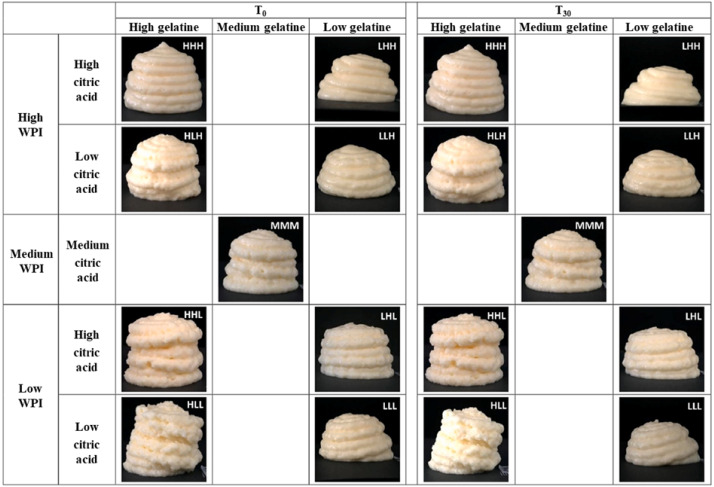	[45]
Whey protein isolate and gelatin	Yoghurt	The addition of WPI, acting as an inert filler, reduced the gelatin effects on the increase in yield stress and other rheological properties, resulting in a softer and better-shaped 3D-printed yoghurt gel.	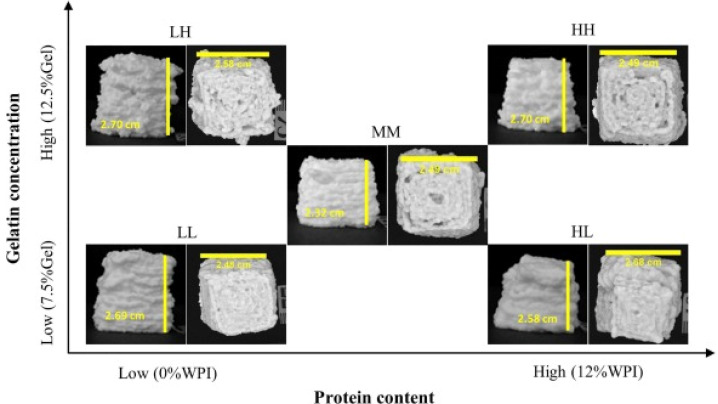	[46]
Insect protein: mealworm protein	Chicken breast	The MPI decreased the viscoelastic properties of surimi ink, showing a loose compact structure, contributing to the soft texture of the printed surimi, which can be applied to the elderly diet.	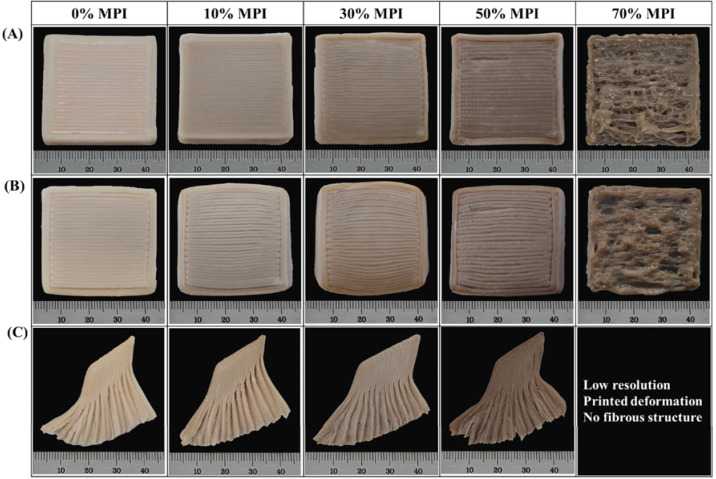	[47]

*Note.* The figures from the references were used in the Table 1, with the copyright from MDPI and Elsevier.

**Table 2:** Approaches to the design of 3D-printed food structures to meet the needs of the elderly (adapted from Calligaris et al. [90]).

Design Direction	Design Details	Examples
Improving proteins	Processing interventions; intaking of various sources proteins	A printed product based on rice, soya protein, and gluten [59].
Carbohydrate limitation	Processing interventions; interaction with other ingredients	A 3D-printed cheesecake with added guava, green tea, and barley sprouts had a GI value of 35.23–56.49% [68].
Salt and sugar reduction initiative	Modulation of sensory perception	Personalized texture sweetmeats were presented using polyol-addition formulations with physical qualities suited for 3D-printing and good nutritional content (low-calorie sweetmeats compared to sucrose-based market-available sweets) [91].
Balancing the consumption of saturated and unsaturated fatty acids	Adding oleogel; using emulsions as fat substitutes	Construction of 3D printable Pickering emulsion gels using complexes of fiber polysaccharide protein extracted from *Haematococcus pluvialis* residues with gelatin as a fat substitute [89].
Increasing fiber intake	Application of physical and/or enzymatic treatments	The 3D-printed cod protein composite gel with inulin and soy dietary fiber [73].
Improving intake of vitamins, minerals, and bioactive compounds	In vivo/vitro delivery to the small intestine	Grape juice gels deactivated NF-κΒ (nuclear factor kappa Β) after simulated gastrointestinal digestion [92].
Improving water consumption	Structuring liquid foods	3D-printed grape juice gels made from gelatin/starch to reduce choking risk [92].
Steering oral and digestion processing	Destructuring hard fibrous foods; design to simulate the digestive system of the elderly	The swallowability of the 3D-printed mooncake was improved by adding soybean oil and Arabic gum [93].
Delivering probiotic bacteria	In vitro delivery system design	The Pickering emulsion made by tea protein/xanthan gum was effective in protecting probiotics, enhancing the design stability of *Bifidobacterium lactis* [78].

## Data Availability

Not applicable.
